# A novel motif in the NaTrxh N-terminus promotes its secretion, whereas the C-terminus participates in its interaction with S-RNase *in vitro*

**DOI:** 10.1186/1471-2229-14-147

**Published:** 2014-05-28

**Authors:** Alejandra Ávila-Castañeda, Javier Andrés Juárez-Díaz, Rogelio Rodríguez-Sotres, Carlos E Bravo-Alberto, Claudia Patricia Ibarra-Sánchez, Alejandra Zavala-Castillo, Yuridia Cruz-Zamora, León P Martínez-Castilla, Judith Márquez-Guzmán, Felipe Cruz-García

**Affiliations:** 1 Departamento de Bioquímica, Facultad de Química, Universidad Nacional Autónoma de México, Ciudad Universitaria, México 04510, Distrito Federal, México; 2 Departamento de Biología Comparada, Facultad de Ciencias, Universidad Nacional Autónoma de México, Ciudad Universitaria, México 04510, Distrito Federal, México

**Keywords:** Thioredoxin, Secretion, Self-incompatibility, *Nicotiana alata*, Gametophytic, S-RNase

## Abstract

**Background:**

NaTrxh, a thioredoxin type *h*, shows differential expression between self-incompatible and self-compatible *Nicotiana* species. NaTrxh interacts *in vitro* with S-RNase and co-localizes with it in the extracellular matrix of the stylar transmitting tissue. NaTrxh contains N- and C-terminal extensions, a feature shared by thioredoxin *h* proteins of subgroup 2. To ascertain the function of these extensions in NaTrxh secretion and protein-protein interaction, we performed a deletion analysis on NaTrxh and fused the resulting variants to GFP.

**Results:**

We found an internal domain in the N-terminal extension, called Nβ, that is essential for NaTrxh secretion but is not hydrophobic, a canonical feature of a signal peptide. The lack of hydrophobicity as well as the location of the secretion signal within the NaTrxh primary structure, suggest an unorthodox secretion route for NaTrxh. Notably, we found that the fusion protein NaTrxh-GFP(KDEL) is retained in the endoplasmic reticulum and that treatment of NaTrxh-GFP-expressing cells with Brefeldin A leads to its retention in the Golgi, which indicates that NaTrxh uses, to some extent, the endoplasmic reticulum and Golgi apparatus for secretion. Furthermore, we found that Nβ contributes to NaTrxh tertiary structure stabilization and that the C-terminus functions in the protein-protein interaction with S-RNase.

**Conclusions:**

The extensions contained in NaTrxh sequence have specific functions on the protein. While the C-terminus directly participates in protein-protein interaction, particularly on its interaction with S-RNase *in vitro*; the N-terminal extension contains two structurally different motifs: Nα and Nβ. Nβ, the inner domain (Ala-17 to Pro-27), is essential and enough to target NaTrxh towards the apoplast. Interestingly, when it was fused to GFP, this protein was also found in the cell wall of the onion cells. Although the biochemical features of the N-terminus suggested a non-classical secretion pathway, our results provided evidence that NaTrxh at least uses the endoplasmic reticulum, Golgi apparatus and also vesicles for secretion. Therefore, the Nβ domain sequence is suggested to be a novel signal peptide.

## Background

Thioredoxins (Trxs) are widely distributed in nature from prokaryotes to eukaryotes. These proteins, which belong to the oxidoreductase thiol:disulfide superfamily [1], are characterized by the active site signature sequence WCXXC. This sequence motif constitutes the redox center mediating the isomerization of specific disulfide bridges on Trx target proteins [2]. In yeasts and mammals, the cytoplasmic Trx redox system is complemented by a second Trx system within mitochondria. In plants, the system is more intricate due to the presence of chloroplastic Trxs that are strongly associated with the regulation of chloroplast metabolism and function [3]. In mammals and yeast, only two and three Trx-encoding genes, respectively, have been identified so far. In contrast, about 19 genes encoding Trxs are contained in *Arabidopsis thaliana* genome, recently reviewed in [4,5].

Trxs were initially described as reductants of ribonucleotide reductase during DNA synthesis [6,7]. Later, these proteins were shown to take part in a variety of important physiological processes, for example as electron donors for several biosynthetic oxidoreductases [8-10] or as protectants against oxidative damage by reduction of the disulphide bridges within many proteins. Interestingly, Trxs and Trx-related proteins are being found to be involved in several sexual plant reproduction processes as well, as reviewed in [11]. The functional diversity of Trxs correlates with their wide distribution in nature and with the large variability in their primary structures (from 27% – 69% of identity among the amino acid sequences) [12]. Their features and functions have been recently reviewed [13,14].

Plant Trxs can be divided into eight types based on their sequence [15]. Types *f*, *m*, *x, y*, and *z* are localized in chloroplasts, type *o* is found in mitochondria, and type *s* is associated with the endoplasmic reticulum (ER) [2,15-19]. Information about the subcellular localization of type *h* (Trxs h), the largest group of this protein family, is limited since this group includes proteins located in the cytosol as well as in mitochondria and even secreted to the apoplast [20-22].

Plant Trxs are also involved in highly specialized biological processes, including self-incompatibility (SI) in *Brassica*[23]. Two Trxs h proteins, THL1 and THL2, interact with the C-terminal domain of the *S*-locus receptor kinase (SRK), which is the female determinant in the sporophytic SI system in *Brassica*[24]*.* The formation of the SRK-THL complex occurs during self-compatible pollinations and it has been proposed that it prevents the SRK dimerization and self-phosphorylation; the last event is essential to the activation of the pollen rejection response [23]. Moreover, suppression of THL1 and THL2 in transgenic plants has shown that both Trxs are required for full pollen acceptance [25]. Trxs h also may play a role in the gametophytic S-RNase-based SI system in *Nicotiana alata* since NaTrxh reduces *in vitro* to the S-RNase, the female *S*-determinant [22]. Moreover, the NaTrxh transcript is more abundant in SI species than in self-compatible ones from *Nicotiana* spp. [26]. In general, evidence indicating the involvement of Trxs and, in general, thiol/disulphide containing proteins within plant sexual reproduction processes is increasing, meaning that redox regulation plays a pivotal role in regulating these signalling mechanisms [11].

Trx h group is subdivided into three subgroups [27]. Subgroup 2 includes Trxs with an N-terminal extension. Some evidence suggests a role for this extension in Trx intracellular trafficking. In *Populus tremula*, the N-terminus of PtTrx*h*2 functions as a mitochondrial targeting signal [21]. As with other subgroup 2 members, *N. alata* NaTrxh contains extensions toward its C- and N-termini, but their functions have not been investigated. Notably, NaTrxh does not possess a canonical signal peptide at its N-terminus but is secreted onto the extracellular matrix of the style [22]. Therefore, either or both the N- or C-terminus could be involved in NaTrxh secretion and/or mediate the protein-protein interaction of NaTrxh with its target proteins.

Here, we show that NaTrxh secretion depends on an inner segment within its N-terminal extension. This segment, Nβ, guides secretion of NaTrxh through the ER and Golgi. In addition, pull-down assays indicate that the C-terminal extension participates in the interaction with S-RNase. Likewise, *in silico* structure modeling predicts both the N- and C-terminal extensions to be solvent exposed and to fold into stable secondary structure elements. The model is consistent with an active role of both extensions in tertiary structure stabilization, with little or no effect on NaTrxh reductase activity.

## Results

### NaTrxh localizes to the extracellular matrix of the transmitting tissue in *N. alata* styles or associates with secretory pathway elements

**Figure 1:**
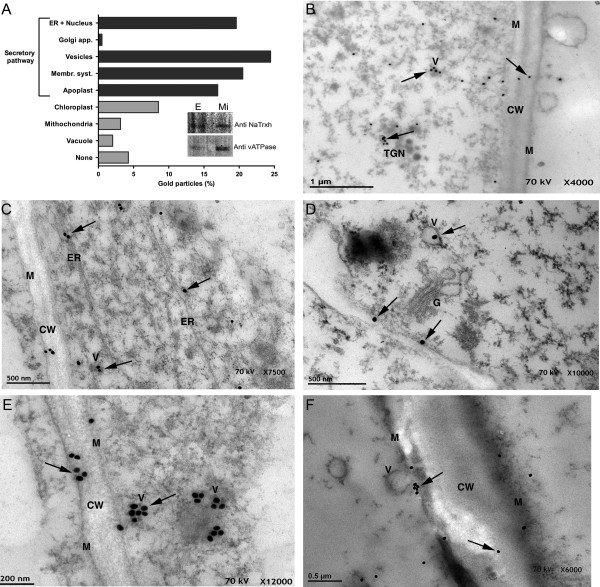
**NaTrxh localized to the cell wall or associates to secretory elements in *N. alata *styles. (A)** Semi-quantitative analysis of the localization of the gold particles (i.e., NaTrxh) by the electron microscopic immune-gold assays. Sub-panel shows NaTrxh was immunodetected in a stylar microsomal fraction (Mi) along with vATPase. E: crude protein extract. **(B)** NaTrxh was associated with vesicles (V), the trans-Golgi network (TGN), or in the cell wall (CW). **(C – D)** NaTrxh was mainly found associated to membranous systems, such as the endoplasmic reticulum (ER), the Golgi apparatus (G), or within vesicles. **(E – F)** Vesicles containing gold particles. In (f), a vesicle is observed fused to the plasma membrane (M). NaTrxh localization (arrows). Scale bars are shown in each micrograph. **(B – F)** Ultra thin sections of *N. alata* styles were treated with anti-NaTrxh and then with anti-rabbit coupled to gold particles.

Previously, we demonstrated that NaTrxh co-localizes to the extracellular matrix (ECM) of the stylar transmitting tissue in *N. alata* along with the S-RNase [22]. Although it lacks a canonical signal peptide, NaTrxh contains sufficient information to guide its secretion, raising the possibility that this protein could follow a non-classical secretion pathway, as suggested by the Secretome 1.0 algorithm [22]. Immuno-gold labelling and electron microscopy data were consistent with an NaTrxh localization at the ECM of the same *N. alata* stylar tissue (Figure 1). Notably, a semi-quantitative analysis, counting all observed particles from five different micrographs at a 12 K resolution, revealed gold particles to be associated with structures related to the secretory system (Figure 1A). This association is consistent with the immune detection of both NaTrxh and the vATPase (marker) in the microsomal fraction of a protein crude extract from *N. alata* styles (Figure 1A, sub-panel). In Figure 1B, D, E, and F, gold particles (i.e., NaTrxh) are observed in association with vesicles, some of which reach the plasma membrane. These images are suggestive of membrane fusion leading to the extracellular release of the vesicle content, including NaTrxh (Figure 1F), which also was found at the ECM, labelled as cell wall (CW; Figure 1B, C, E, F). Figure 1C and D are representative micrographs where NaTrxh was found in association either with the ER or the Golgi. In contrast to the Secretome 1.0 algorithm prediction [22], our data show at least a fraction of NaTrxh travelling through the ER and Golgi secretory pathway en route to its final apoplastic localization in the styles of *N. alata*. However, as previously mentioned, NaTrxh lacks a canonical signal peptide, and the localization found through immuno-gold and electron microscopy provides cellular confirmation of secretion.

### NaTrxh N- and C-terminal extensions

**Figure 2:**
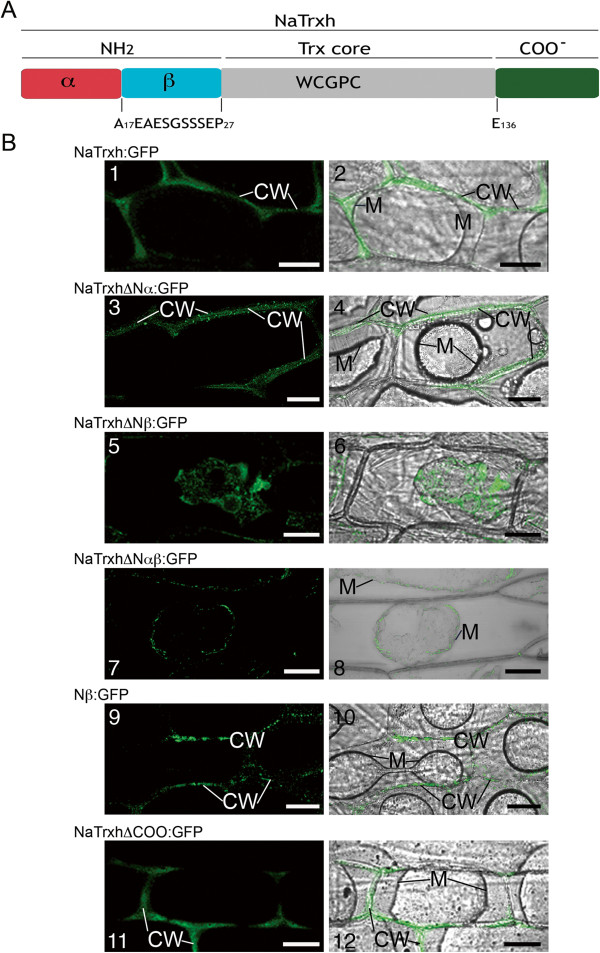
**The Nβ motif is responsible for NaTrxh secretion. (A)** An NaTrxh scheme indicating its N- and C-terminal extensions. The N-terminus was subdivided into two regions: Nα (red) and Nβ (cyan). The C-terminus (green). **(B)** Transient expression of the different NaTrxh mutants fused to GFP in onion epidermal cells. **(B-1) (B-2)** Full-length NaTrxh. **(B-3) (B-4)** NaTrxhΔNα. **(B-5) (B-6)** NaTrxhΔNβ. **(B-7) (B-8)** NaTrxhΔNαβ. **(B-9) (B-10)** Nβ motif (Ala-17 to Pro-27) directly fused to GFP. **(B-11) (B-12)** NaTrxhΔCOO. **(B-1) (B-3) (B-5) (B-7) (B-9) (B-11)** GFP fluorescence. **(B-2) (B-4) (B-6) (B-8) (B-10) (B12)**. Bright fields merged with flourescence images. The cells were plasmolyzed with 1 M NaCl before confocal observation. CW: cell wall; M. plasma membrane. Scale bars = 50 μm.

As previously reported [22] and shown in Figure 1, NaTrxh is secreted in *N. alata* styles. Contrary to the Secretome 1.0 algorithm, which predicts a non-classical secretion signal for NaTrxh, the hidden Markov algorithm [28] predicts a cleavage site between residues Ala-16 and Ala-17, albeit with a low probability (*p = 0.593*) [22]. Multiple alignment of several Trxs *h* from subgroup 2 showed that the NaTrxh N-terminal extension sequence is at least 27 residues long (Additional file 1: Figure S1) and its C-terminal extension comprises residues E-136 to Q-152 (Additional file 1: Figure S1).Based on the above predictions, we divided the N-terminus of NaTrxh in two motifs: Nα (from Met-1 to Ala-16) and Nβ (Ala-17 to Pro-27). The C-terminal extension was defined starting at E-136 (Figure 2A).

### The Nβ region is crucial for NaTrxh secretion

To test if either extension is responsible for NaTrxh secretion, we generated NaTrxh deletion mutants lacking different sequence segments, fused to green fluorescent protein (GFP), and then transiently expressed them in onion epidermal cells.

**Figure 3:**
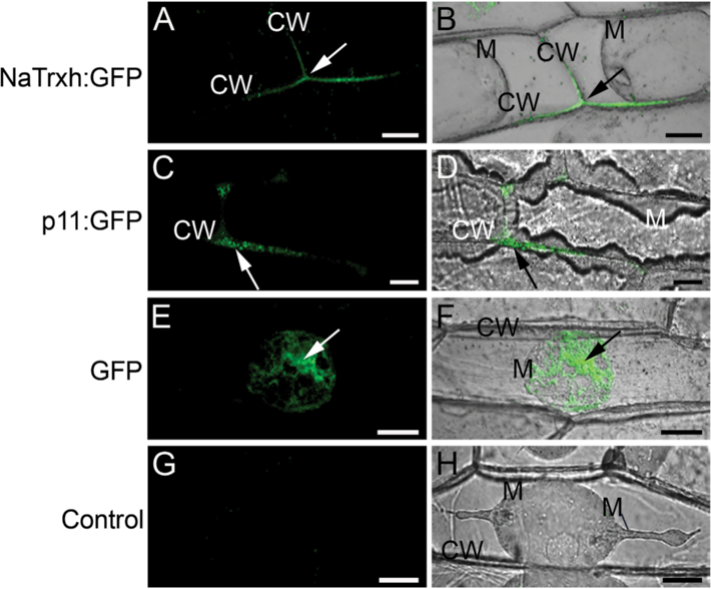
**NaTrxh: GFP is secreted in onion epidermal cells. (A – B)** GFP fluorescence from the NaTrxh: GFP fusion protein, was localized on the cell wall (CW). **(C – D)** p11 is a known secreted protein in *N. alata* styles that was also secreted. **(E – F)** GFP alone was not secreted when transiently expressed. **(G – H)** Non-transformed cells. **(A, C, E, G)** GFP fluorescence. **(B, D, F, H)** Bright fields merged with flourescence images. M: plasma membrane; CW: cell wall; GFP fluorescent signal (arrows). The cells were plasmolized with 1.0 M NaCl before confocal observation. Scale bars = 30 μm.

**Figure 4:**
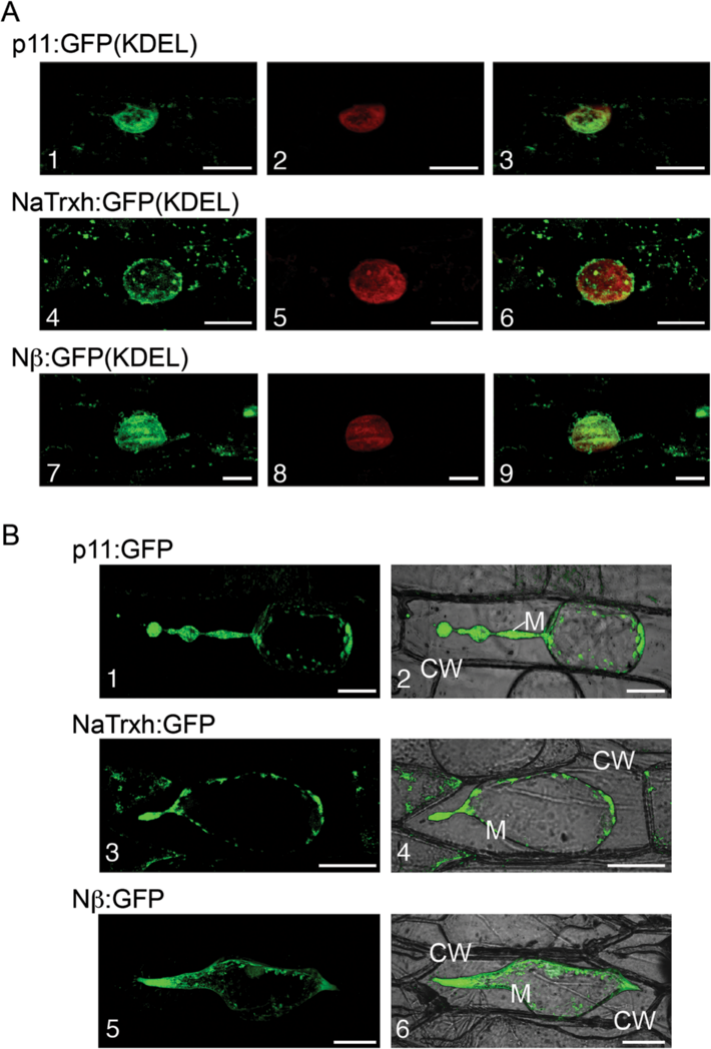
**NaTrxh uses the ER/Golgi secretion elements to reach the apoplast. (A)** Transient expression in onion cells of different proteins with the ER retention signal (KDEL) toward the C-termini. **(A-1) (A-4) (A-7)** GFP fluorescence. **(A-2) (A-5) (A-8)** Nucleus staining with propidium iodide. **(A-3) (A-6) (A-9)** Merged images. Scale bars = 20 μm. **(B)** Transient expression of p11:GFP, NaTrxh:GFP and Nβ: GFP in onion cells treated with BFA (50 μg/ml). **(B-1) (B-3) (B-5)** GFP fluorescence. **(B-2) (B-4) (B-6)** Bright fields merged with fluorescence images. The observations were made after plasmolysis with 1 M NaCl. CW: cell wall; M. plasma membrane. Scale bars = 50 μm.

**Figure 5:**
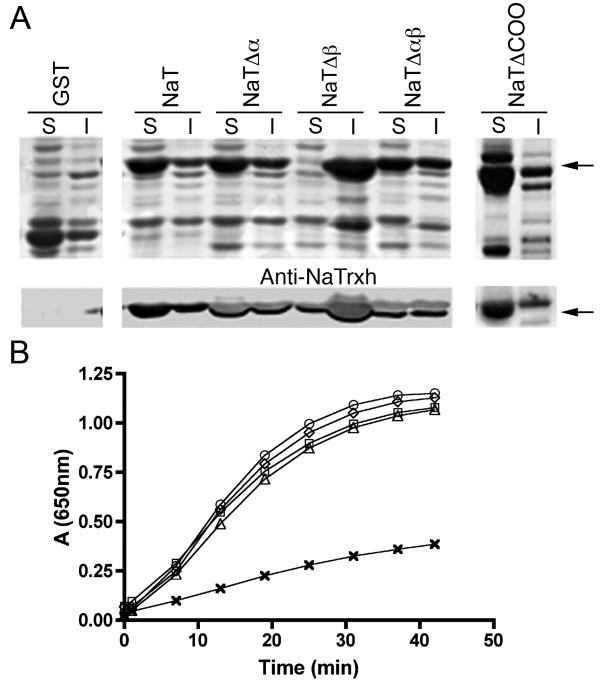
**Nβ domain contributes to NaTrxh stability. N- and C-termini are not involved in its reductase activity. (A)** SDS-PAGE analysis of different NaTrxh versions fused to GST expressed in *E. coli* cells. Upper panels: Coomassie blue stained gel; lower panels: western-blot immuno-stained with anti-NaTrxh antibody. S: soluble fraction; I: insoluble fraction; GST: gluthathione S-transferase; NaT: full-length NaTrxh; NaTΔα: NaTrxhΔNα mutant; NaTΔβ: NaTrxhΔNβ; NaTΔαβ: NaTrxhΔNαβ; NaTΔCOO: NaTrxhΔCOO. Arrows indicate the signal corresponding to the different NaTrxh:GST versions. **(B)** Thioredoxin activity assay using insulin as substrate and DTT as electron donor (Holmgren, 1979). Circles: NaTrxh; Diamonds: NaTrxhΔNα; Squares: NaTrxhΔNαβ; Triangles: NaTrxhΔCOO; Crosses: only DTT.

**Figure 6:**
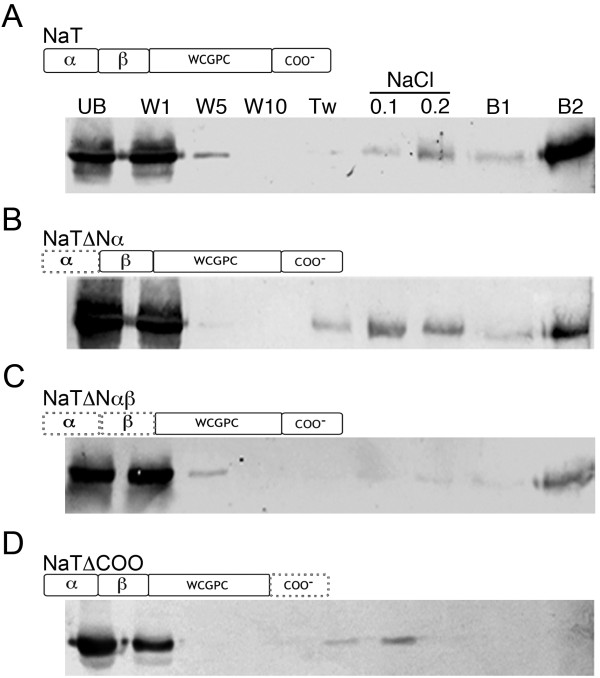
**The NaTrxh C-terminus contributes to the NaTrxh: S-RNase interaction.** Pull-down experiments were performed using columns with the different NaTrxh versions [**(A)** NaT: NaTrxh; **(B)** NaTΔα: NaTrxhΔNα; **(C)** NaTΔαβ: NaTrxhΔNαβ; **(D)** NaTΔCOO: NaTrxhΔCOO]. Dotted lines indicate deleted regions. Stylar proteins from *S*_105_*S*_105_*N. alata* were passed through each column, and then, each recovered fraction was analysed by western blot immune-staining with anti-S_105_-RNase. UB: unbound fraction; W1, W5, and W10: first, fifth, and tenth washes, respectively, with binding buffer; Tw: binding buffer plus 0.1% Tween-20; NaCl 0.1 and 0.2: washes with 50 mMTris, pH 7.9 + 100 mM or 200 mM NaCl, respectively; B1 and B2: bound fractions eluted with one and two bed volumes of elution buffer.

**Figure 7:**
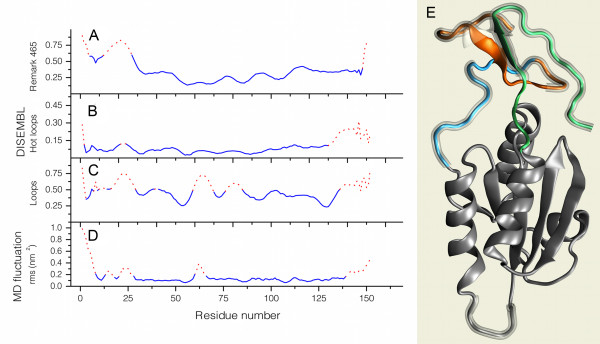
**N- and C-terminal extensions are predicted to be disordered and solvent exposed. (A – C)** Plots of DisEMBL Remark 465, hot loops, and loop index values. Red dashed lines indicate amino acid positions over the default threshold. **(D)** RMSD fluctuation average per amino acid for the backbone atoms of the NaTrxh model during the last 2.5 ns of MD simulation. **(E)** Cartoon of the NaTrxh final model relaxed with ROSETTA fast-relax. Segments were colored according to Figure 2A. The glassy shades indicate areas predicted as highly mobile, according to the plot in **D**; image prepared with VMD [36].

First, we showed that the full-length NaTrxh fused to GFP is observable in the extracellular space of onion epidermal cells (Figure 3A and B), as reported in *N. benthamiana* and *A. thaliana*[22]. The same was observed for the stylar ECM protein p11 [29] fused to GFP (Figure 3C and D). We observed the same pattern when the Nα motif is deleted from the N-terminus of NaTrxh (NaTrxhΔNα: GFP; Figure 2B-3 and 2B-4) and, therefore, concluded the Nα domain is not required for targeting NaTrxh to the apoplast. However, when NaTrxhΔNαβ, which lacks both the Nα and Nβ motifs, was expressed as a GFP fusion protein, fluorescence was localized inside the cells, indicating that secretion was abolished (Figure 2B-7 and B-8). When the C-terminus was deleted from NaTrxh (NaTrxhΔCOO: GFP), GFP fluorescence was localized to the apoplast (Figures 2B-11 and B-12). These data show that the N-terminal extension carries all the information for NaTrxh secretion. However, in contrast to an orthodox N-terminal signal peptide, the first 17 amino acids are not required, as the inner Nβ domain promotes secretion in the absence of the Nα segment. To test this hypothesis, we generated an NaTrxh protein mutant with the Nα domain adjacent to the Trx core, deleting the Nβ domain (NaTrxhΔNβ), and then expressed it as a GFP fusion protein. Transient expression of NaTrxhΔNβ: GFP is shown in Figures 2B-5 and B-6. GFP fluorescence can be observed within the cytosol. Furthermore, fusion to GFP of the Nβ domain alone leads to extracellular localization of the GFP signal, which resembles the distribution found for full-length NaTrxh (Figure 2B-9 and B-10). Together, these outcomes provide strong evidence that the Nβ domain is both essential and sufficient for NaTrxh secretion.

### NaTrxh uses the endomembrane system to reach the apoplast

While clearly sufficient to function as a secretion signal, the Nβ domain may guide NaTrxh secretion through an unorthodox secretion pathway. This possibility is suggested by the Nβ domain’s unusual position within the primary structure (17 residues from the N-terminus) and the absence of a long hydrophobic amino acid region (Additional file 2: Figure S2). To evaluate if Nβ-led secretion proceeds via the ER, we looked for the presence of NaTrxh in the ER using two NaTrxh fusion proteins, NaTrxh:GFP(KDEL) and Nβ: GFP(KDEL), both of which exhibit the ER retention signal KDEL [30,31]. As a control, we also fused p11 to GFP(KDEL). p11 is a known secreted protein from *N. alata*[29] with a typical signal peptide that is expected to follow the classical ER/Golgi pathway. The GFP signal from all GFP(KDEL) fusion proteins exhibits a typical ER distribution pattern surrounding the nucleus. The reticulate fluorescent pattern observed with both fusion proteins (Figure 4A-1 and A-4) and, interestingly, with the Nβ: GFP(KDEL) as well (Figure 4A-7), contrasts with the blurred pattern of the nucleus (Figures 4A-2, A-5 and A-8). These data are consistent with the passage of NaTrxh through the ER on its way out of the cell (Figure 4A).Evidence for participation of the Golgi network in NaTrxh secretion was obtained from treatment of onion epidermal cells with the fungal toxin Brefeldin A (BFA). BFA blocks vesicle formation at the Golgi network, which prevents secretion of Nap11:GFP, NaThx:GFP, and Nβ:GFP (Figure 4B). Additional evidence that NaTrxh is secreted through vesicles is NaTrxh association with a membrane fraction (Figure 1A). Taken together, these results show that the Nβ domain is a hydrophilic novel internal signal able to promote NaTrxh secretion via the ER/Golgi.

### The N-terminal region of NaTrxh accounts for structural stability but not for its reductase activity

To evaluate whether the N-terminal extension, the C-terminal extension, or both extensions participate in NaTrxh reductase activity, we overexpressed four NaTrxh mutants as GST fusion proteins in *Escherichia coli*. The NaTrxhΔNα, NaTrxhΔNαβ, and NaTrxhΔCOO proteins were recovered from the soluble phase from bacterial sonicates (Figure 5A), as reported for the full NaTrxh [22]. Notably, NaTrxhΔNβ is only detected at the insoluble phase (Figure 5A), suggesting that the protein does not fold correctly; therefore, its activity as a disulphide reductase could not be tested. When compared to full-length NaTrxh, the NaTrxh variants show no differences in their ability to reduce insulin disulfide bonds using dithiothreitol (DTT) as an electron donor (Figure 5B) [7]. This result demonstrates that the N-terminal extension functions in NaTrxh trafficking and, like the C-terminus, does not participate in NaTrxh’s ability to reduce target proteins.

### *N. alata* S-RNase interacts *in vitro* with NaTrxh by its C-terminal region

We previously reported the *in vitro* interaction of NaTrxh with the pistil *S*-determinant S-RNase from *N. alata*. The interaction takes place regardless of the NaTrxh redox state [22]. To test whether the N-terminal or C-terminal region accounts for this specific protein-protein interaction, we prepared GST:NaTrxh-, GST:NaTrxhΔNα-, GST:NaTrxhΔNαβ-, and GST:NaTrxhΔCOO-Affi-Gel affinity columns and passed through them extracellular stylar protein extracts from *N. alata S*_105_*S*_105_.

Figure 6A shows that the S_105_-RNase was retained in the NaTrxh-GST-Affi-gel matrix, as reported by Juárez-Díaz et al. [22]. Notably, we observed a similar binding behaviour when crude style extracts from *N. alata S*_105_*S*_105_ were passed through the NaTrxhΔNα and NaTrxhΔNαβ matrices (Figure 6B and C). Noteworthy, when the protein extracts are passed through the affinity column with NaTrxhΔCOO, the S_105_-RNase is not retained (Figure 6D). These data show that the NaTrxh C-terminus contributes to the interaction with the S_105_-RNase.

### The Nβ domain plays a structural role in NaTrxh

NaTrxh is predicted to interact with other trafficking-related proteins to be secreted. Thus, the Nβ domain is likely to be exposed at the molecular surface to facilitate such interactions. To support this hypothesis, we constructed a model of NaTrxh using a combination of homology modeling and molecular dynamic (MD) simulations. We used Modeller 9v4 [32] for the homology modelling and GROMACS 3.3.1 [33,34] for the MD simulations.

While the closest homologue of NaTrxh with a known 3D-structure is the *Hordeum vulgare* H2 Trx (2IWT), the *N. alata* protein possesses extensions toward its N- and C-termini, which has no homologues in the Protein Data Bank (PDB) [35]. We obtained a predicted conformation for these extensions by performing two rounds of MD simulations. The structure shown in Figure 7E is a representative conformation, drawn with visual molecular dynamics (VMD) molecular viewer [36]; mobile regions are shown in orange, blue and green. At the end of the second run, the N- and C-termini folded to form a “beta sheet hat” separated from the Trx core and opposite the putative reactive site loop (with the motif xCxPCx). The beta sheet was fully formed after 20 ns and remained stable thereafter. Only four segments in the protein showed significant fluctuation in the final model: the first 5 and the last 5 residues, the loop where the reactive cysteine residues reside (60 to 66), and a loop connecting the core to the N-side of the “beta sheet hat” (residues 23 to 26).

The model was rated from *very good* to *fairly good* by Atomic Non-Local Environment Assessment (ANOLEA) [37] (Figure 7B) and ProQ [38]. With the Rd.HMM protocol [39], we used the coordinates of the backbone atoms of the model (after replacement of sequence information with random amino acid sequences) to retrieve the *N. alata* NaTrxh amino acid sequence from the NCBI nr protein database [40] with a statistical significance substantially higher than the one for the *H. vulgare* sequence (homology modeling template protein). In contrast, the 2IWT crystal as well as some of the initial models from Modeller, when subjected to the Rd.HMM protocol, scored the sequence of the barley protein and several other Trx h proteins with high probability, while the *N. alata* amino acid sequence was recovered with an E value above 1 (lacking statistical significance). According to its quality and appropriateness scores (see Methods), the model appears to be reasonably close to the reduced form of the *N. alata* NaTrxh 3D structure. The appropriateness score is worth noting because the Rd.HMM is known to be very sensitive, which may result in false negatives (i.e., the model is rejected even when it may be an approximate description of the native-like 3D structure). However, no false positives have been found yet.

Interestingly, in all models produced, the N-terminus remains accessible to the solvent, especially the region corresponding to residues 20 to 28, which coincides with the Nβ domain. In addition, the final conformations of the N- and C-termini anchors and the N-terminal extension to the hat (Figure 7E) forces the poorly ordered loop of amino acids from residues 23 to 26 to remain on the protein surface. Since this region seems to be sufficient for NaTrxh secretion, its anchorage may facilitate the recognition of this sequence by some unidentified component of the secretion pathway. To assess the potential of the NaTrxh extensions to interact with other proteins, we compared them to intrinsically disordered regions (IDRs). The amino acid sequences known as intrinsically disordered proteins (IDPs) or IDRs, among other names, are proteins or partial regions of proteins that lack stable and well-defined 3D structures under physiological conditions *in vitro*[41-43]. We identified the IDRs using DisEMBL [28]; server at [44], which relies on three criteria to assign an amino acid sequence as disordered: loops/coils, hot loops, and remark465. The loops/coils definition identified residues 1 to 47, 59 to 70, 75 to 86, and 135 to 152 as IDRs (Figure 7C); hot loops reported segments 19 to 26 and 118 to 152 as IDRs (Figure 7B); and remark465 defined the first 28 N-terminal residues as the only IDR in NaTrxh (Figure 7A). According to DisEMBL, the NaTrxh extensions are IDRs, and all three criteria agree with MD simulations in the prediction of the Nβ region (Figure 7D) as a poorly structured protein segment.

## Discussion

Here, we demonstrated that the Nβ domain (A_17_EAESGSSSEP_27_) is required for NaTrxh secretion. Additionally, we provided evidence on NaTrxh targeting to the apoplast via the ER/Golgi regardless of the absence of a distinguishable hydrophobic signal peptide. Finally, we also present data that suggest that the C-terminal region of NaTrxh is an important mediator of the NaTrxh:S-RNase interaction.

### NaTrxh is transported through vesicles toward the apoplast

The immune assays we performed clearly show that NaTrxh is mainly localized to membranous bodies, primarily vesicles, which correlate with the finding of NaTrxh in the microsomal fraction. These data strongly indicate that NaTrxh is carried to the extracellular space by means of a vesicle-dependent secretion pathway. The electron microscopy data clearly place NaTrxh inside vesicles (Figure 1), although some gold particles were observed to be associated with ER and other unidentified membranous systems, we cannot affirm that these vesicles come from the ER, the Golgi, or both.

Although NaTrxh lacks a canonical signal peptide, its association with vesicles correlates well with its extracellular localization. A possible secretion mechanism for proteins of this kind relies on their direct interaction with secretion vesicles without previous association to the ER/Golgi [45]. In mammalian cells and yeast, some proteins are secreted through an ER/Golgi-independent pathway. Such is the case of insulin degrading enzymes [46], interleukins IL-1b and IL-18 [47], and some yeast proteins lacking a signal peptide [45].

### NaTrxh has an internal and hydrophilic secretion signal and is secreted via the ER/Golgi

The symplastic localization of the NaTrxhΔNα mutant (Figure 2B-3, B-4) shows that the first 17 N-terminal residues are not essential for NaTrxh secretion. Instead, the internal amino acid sequence A_17_EAESGSSSEP_27_ (Nβ), despite lacking the characteristic hydrophobicity (Additional file 2: Figure S2) shown on classical signal peptides [48], is essential and sufficient for NaTrxh secretion, as observed by the cytoplasmic localization of the NaTrxhΔNβ mutant (Figures 2B-5, B-6). This motif is also sufficient to direct the Nβ GFP-tagged to the extracellular space (Figures 2B-9, B-10). Most proteins secreted through the ER/Golgi pathway are translated in ribosomes attached to the ER membrane and possess a signal peptide localized at the N-terminus [48]. One important property of such signal peptides is their hydrophobicity [48]. This feature is essential for recognition of the nascent peptide by the signal receptor particle (SRP) [49]. Although our data indicate that NaTrxh passes through the ER and Golgi en route to the apoplast —as shown with the KDEL constructs (Figure 4A), the blocking of NaTrxh secretion by BFA (Figure 4B), and the presence of NaTrxh in the microsomal fraction (Figure 1A)— we do not know how NaTrxh is transported into the ER and how the Golgi participates in its secretion. Although several possible scenarios are feasible, we currently have no evidence to favor any of them. Two examples that support secretion of proteins without a conventional signal peptide and using the endomembrane system are the proteins IL-1β and AcbA (acyl-coenzyme A-binding protein) [50]. IL-1β joins secretory lysosomes and is released when those lysosomes fuse with the plasma membrane [51,52]. IL-1β also can be captured directly into multivesicular bodies or be sequestered by autophagosomes and fuse with multivesicular bodies [52,53].

Non-classical secretion of cytoplasmic plant proteins has also been documented, as reviewed in [54,55]. It has been demonstrated that proteins without signal peptide, such as celery mannitol dehydrogenase in *A. thaliana*, traffic to the apoplast while bypassing the classical ER/Golgi secretion pathway [56]. Another example is the hygromycin phosphotransferase in *A. thaliana*, which is secreted through a Golgi-independent route mediated by the Golgi-localized synaptotagmin 2 [57]. However, this is unlikely to be the case for NaTrxh since our data clearly showed that it goes through both ER and Golgi for its secretion (Figure 4).

Another possible route that NaTrxh could follow to the apoplast is through specialized vesicles, such as the exosome-like nanovesicles described in *Olea europea* pollen tubes, called pollensomes [58]. Some of the pollensomes are proposed to be ER- and Golgi-derived vesicles based on the fact that Ole e 1 from *O. europea* was found to be within these pollensomes [58]. Regarding NaTrxh, we observed that some of it was contained in cytoplasmic vesicles and some of them were observed fused to the plasma membrane (Figure 1E and F). In the apoplast, NaTrxh was never found associated to any exosome-like structure, as described for pollensomes [58] (Figure 1).

An additional possibility is that NaTrxh could associate to endomembrane systems through lipidic modifications. Actually, Traverso et al. [59] found that NaTrxh is *in vitro* myristoylated at Gly-2, suggesting that NaTrxh may be a membrane-associated protein *in planta.* Based on this, it was speculated that it could be the manner about how NaTrxh is transported to the apoplast [59,11]. However, this scenario appears to be unlikely to occur because our deletion analysis outcomes indicated that the first 16 amino acids (the Nα motif) are not essential for NaTrxh secretion, instead it was the inner domain, the Nβ motif, the one that directly led its secretion (Figure 2).

The Nβ motif is apparently exclusive to plant Trxs. Besides NaTrxh, a similar motif has been found in only two soybean thioredoxins (Trxh2 and Trxh1) that, notably, are associated with the plasma membrane. Both soybean Trxs have an N-terminal extension [60] that includes a region with a high similarity index to the Nβ sequence (Additional file 3: Figure S3).

Our cell biology data along with our molecular assays by transient expression of different versions of NaTrxh fused to GFP indicate that NaTrxh secretion is due to its Nβ motif and that the protein follows a secretion pathway that requires the ER, the Golgi apparatus, and secretion vesicles. How NaTrxh interacts with these secretory elements is not known since the NaTrxh N-terminus does not have any of the typical signal peptide biochemical properties. However, the absence of an orthodox signal peptide in NaTrxh reveals the existence of an alternative secretion mechanism that uses, to some extent, the ER/Golgi secretory pathway. The accurate mechanism that leads NaTrxh secretion needs to be clarified and future research will be of great interest in order to unravel possible novel plant trafficking routes.

### NaTrxh:S-RNase interaction

Protein and mRNA levels of NaTrxh are higher in the styles of SI plants than in self-compatible plants, and S-RNase interacts with NaTrxh *in vitro*. These facts have been used to classify *NaTrxh* as a pistil modifier gene that accounts for pollen rejection in *N. alata*[22,26].This work contributes to our understanding of the molecular mechanism mediating the NaTrxh: S-RNase interaction. The pull-down experiments show that the NaTrxh C-terminal extension (E-136 to Q-152) is essential for its interaction with S-RNase (Figure 6D). However, this region does not affect NaTrxh secretion (Figure 2B-11, B-12) or Trx activity (Figure 5B). Therefore, it appears that NaTrxh is able to fold correctly in the absence of the C-terminal domain or at least fold well enough to sustain its native-like reductase activity.

In *N. alata*, several proteins are directly involved in pollen rejection. In this species, S-RNase degrades the pollen tube RNA and determines sexual incompatibility on the female side. The NaTrxh:S-RNase interaction could be relevant to the SI response. NaTrxh likely stabilizes S-RNase or inhibits its ribonuclease activity in the pollen tube. Indeed, Oxley and Bacic [61] showed that S-RNase ribonuclease activity is affected by redox state *in vitro*. However, the redox state of NaTrxh does not impair its interaction with S-RNase [22].

S-RNase forms complexes with other stylar proteins (i.e., 120 K, p11, NaTTs) [62]. While 120 K is known to be essential for SI in *N. alata*[63], the precise function of these protein complexes in SI is still unclear. It is possible that NaTrxh participates as an associating factor to transport such as S-RNase, 120 K, NaTTs or p11 to the pollen tube or, alternatively, to release these proteins from S-RNase complexes once inside the pollen tube. Both scenarios may be possible since a redox change by NaTrxh could play an important role for modifying S-RNase and stylar protein complexes. It has been determined that one of the targets of NaTrxh is actually S-RNase [22]. Therefore, further research is needed to determine if NaTrxh is a modifier factor in *N. alata* SI by altering the S-RNase redox state *in planta*. Although the data presented here are consistent with a role of NaTrxh in pollen rejection in SI *Nicotiana* species, loss of function assays would provide direct evidence of this role.

Finally, homology modeling to predict the 3D structure of Trx h revealed high sequence similarity between the *H. vulgare* and *N. alata* Trxh proteins, including conservation of the reactive site loop. The quality of the predicted model indicates similarity at the structural level too. The barley Trx h protein plays an important regulatory role during seed germination [64], and one of its targets is the barley α-amylase/subtilisin inhibitor, a homologue of the SI modifier *N. alata* NaStEP protein recently described by our group [65]. Both NaStEP and *H. vulgare* inhibitor proteins share extensive structural similarity; the loop equivalent to the one mediating the interaction of the barley proteins is present in NaStEP but is longer and has four cysteine residues instead of two [65]. An interaction of NaTrxh and NaStEP at some point along the physiological events regulating pollen rejection in *Nicotiana* is a possibility worth future consideration.

## Conclusions

Thioredoxins type *h* clustered within the subgroup 2 contain non-conserved extensions towards the N- and/or the C-termini, which function is still unclear. In this work, we showed that the N-terminal extension of NaTrxh, a subgroup 2 Trx h from *N. alata*, contained the sufficient information to lead its secretion towards the apoplast. Interestingly, this extension contains two distinguishable motifs, called Nα and Nβ, divided by the hidden Markov algorithm [28] prediction of a cleavage site on the Ala-16 position. While the Nα domain appeared unlikely to have any function on NaTrxh secretion, the Nβ was the responsible for its particular subcellular localization.

The Nβ domain is the only sequence necessary for NaTrxh secretion. Transient expression experiments in epidermal onion cells of the Nβ-GFP fusion protein revealed its localization in the apoplast, as occurred with the full NaTrxh protein fused to GFP and with the NaTrxhΔNα and NaTrxhΔCOO mutants as well. These data corroborated the Nβ function as a novel signal peptide since its primary structure position and hydrophilic profile do not follow the typical biochemical features of the classical transport sequences.

The Nβ biochemical features suggested an ER/Golgi independent secretion pathway. However, NaTrxh was detected in a microsomal fraction and, furthermore, was immune-detected mainly associated to classical secretory elements when observed within the cells in *N. alata* styles, providing evidences that the NaTrxh could be secreted through the classical ER/Golgi pathway or at least, it uses the elements of this route. This hypothesis was also tested by fusing the ER retention signal KDEL to NaTrxh-GFP and treating the cells, when expressing the fusion protein NaTrxh-GFP, with BFA, separately. In the first case, the NaTrxh-GFP(KDEL) was found associated to the ER and in the latter, the NaTrxh-GFP secretion was abolished, confirming that the NaTrxh actually goes through these organelles in order to be secreted. Furthermore, it was also found that the Nβ domain played an important structural role on the NaTrxh tertiary structure stability since the NaTrxhΔNβ, which only lacks the Nβ domain, was detected in inclusion bodies when overexpressed in *E. coli* cells and not in the soluble one as the wild type and the other NaTrxh versions did (i.e., NaTrxhΔNα, NaTrxhΔNαβ and NaTrxhΔCOO).

Regarding the C-terminus, it was found to be essential for the NaTrxh-S-RNase *in vitro* interaction, since the S-RNase was unable to bind to a NaTrxhΔCOO-containing column.

Finally, the *in silico* analysis showed that the NaTrxh N- and C-termini are solvent exposed, suggesting a protein-protein interaction role. While this function appears to be essential for the S-RNase interaction, it also provided evidences on the Nβ domain, which should interact either with a non-identified secretory element or interact in a different manner as the classical secreted proteins do with SRP. Interestingly, the N-terminal extension clearly showed two structural motifs, which coincide with the Nα and Nβ domains tested in this work.

## Methods

### Plant materials

Self-incompatible (SI) *Nicotiana alata S*_105_*S*_105_ has been described previously [22,66-68].

### GST fusion proteins, overexpression and purification from *E. coli*

The NaTrxhΔNα and the NaTrxhΔNαβ cDNAs were generated with *Bam*-HI and *Eco*-RI flanking sites using 5′-GCGC*GGATCCATG*GCAGAGGCAGAATCAG-3′ and 5′-GC*GGATCCATG*TCGCGTGTGATTG-3′ as sense primers, respectively, and 5′-GCGCGCGG*GAATTC*AATTTATTGGACATGAAA-3′ as the antisense primer for both mutants. For the NaTrxhΔCOO mutant, we used 5′-CGCGC*GGATCC*ATGGGATCGTATCTTTCAA-3′ as the forward primer and 5′-CCG *GAATTC*CCTGTGCTTGAGAATCTTTTTCTCGAG-3′ as the reverse primer with *Bam*-HI and *Eco*-RI sites, respectively. The NaTrxhΔNβ cDNA with *Bam*-HI and *Eco*-RI sites was generated by two sequential PCRs. For the first PCR, the forward primer was 5′-TCGCGTGTGATTGCTTTTCATTCTTCCAAT-3′. The PCR product from the first amplification was used as template for the second PCR, with 5′-*GGATCC*ATGGGATCGTATCTTTCAAGTTTGCTCGGTGGAGGCGCGGCGGAAGCGTCGCGTGTGAT-3′ used as the sense primer. The reverse primer in both amplification steps was the same as that used for the other N-mutants.

For overexpression of the mutant forms of NaTrxh, each cDNA was cloned into pGEX 4 T-2 (pGEX) (Amersham Biosciences) in *E. coli* BL21(DE3)pLysS cells (Stratagene), as was previously described for the wild type NaTrxh [22].

The GST:NaTrxh, GST:NaTrxhΔNα, GST:NaTrxhΔNβ, GST:NaTrxhΔNαβ, and GST:NaTrxhΔCOO fusion proteins were overexpressed in *E. coli* cultures at an OD_600_ of 0.5 – 0.7 by adding 0.1 mM IPTG and incubating for 3 – 5 h at 37°C. The proteins were separated by batch affinity chromatography using glutathione agarose (Sigma).

### Constructs for NaTrxh versions, Nβ and p11 fused to GFP

NaTrxh and NaTrxhΔNβ cDNA with *att*B1 and *att*B2 sites were generated by using the following primers: forward, 5′-GGGG*ACAAGTTTGTACAAAAAAGCAGGCTTC*ATGGGATCGTATCTTTCAAGTTTG-3′; reverse, 5′-GGGG*ACCACTTTGTACAAGAAAGCTGGGTC*TTGGACATGAAATTTAGTTCGATA-3′. The pGEX::NaTrxh and pGEX::NaTrxhΔNβ constructs were used as templates, respectively. The PCR products were cloned into pDONR/Zeo (Invitrogen) by recombination with a BP Clonase (Invitrogen), following the manufacturer’s instructions.

The NaTrxhΔNα, NaTrxhΔNαβ, and p11 cDNAs were generated by PCR using the forward primers 5′-*CACCATG*GCAGAGGCAGAATCAGGA-3′, 5′-*CACCATG*TCGCGTGTGATTGCTTTT-3′ and 5′-*CACCATG*TCAGGAAAACAAGGGTCTGCAATTTTATG-3′, respectively. For the NaTrxhΔNα and NaTrxhΔNαβ clones, the reverse primer was 5′-TTATTGGACATGAAATTTAGTTCGATAATTACTAGCAGC-3′. For p11, the reverse primer was 5′ TTTGTTTGTTAACTTAGCAGTAACTGAAATCTTTTGGCC 3′. All cDNAs were cloned into pENTR/D-TOPO (Invitrogen), following the manufacturer’s instructions.

NaTrxhΔCOOcDNA was cloned into pENTR/D-TOPO by using 5′-CACCTGGGATCGTATCTTTCAAGT-3′ and 5′-TCATTCCCTGTGCTTGAGAATCTT-3′ as sense and antisense primers, respectively.

To generate the *Nβ* sequence, the DNA fragment 5′-*CACCATG*GCAGAGGCAGAATCAGGATCGTCGTCAGAACCG-3′ was aligned with its complement by mixing both primers and incubating at 94°C for 15 min and then for 30 min at room temperature before proceeding with cloning into pENTR/D-TOPO (Invitrogen), following the manufacturer’s instructions.

All *NaTrxh* sequences (including *Nβ*) were transferred by recombination by LR recombinase enzymatic mixture (Invitrogen) to pEarleyGateway103 (C-GFP-HIS) (pEG103) [69], following the manufacturer’s instructions. In the plasmid, the gene of interest is translated with GFP fused to its C-terminus under the control of the 35S cauliflower mosaic virus (35S) promoter.

The *NaTrxh:GFP(KDEL)*, *Nβ:GFP(KDEL)*, and *p11:GFP(KDEL)* constructs were generated by PCR using the corresponding pEG103 construct as a template*.* The forward primers were the same as described above for each construct and the reverse was 5′ TCA*AAGCTCATCTTT*GTGGTGGTGGTGGTGGTGGCTAGC 3′, which encodes the amino acids KDEL fused to the C-terminus of GFP from pEG103.

### Transient expression assays in onion epidermal cells

Each fusion construct *35S*:*NaTrxh-GFP* was individually bombarded into onion epidermal cells [70]. After 24 h of particle bombardment, onion epidermal cells were plasmolyzed by incubation in 1 M NaCl for 10 min. Fluorescence was visualized using an Olympus FV 1000 confocal microscope with 485/545 nm excitation/emission light for GFP and 570/670 nm for propidium iodine (Sigma), which was used to stain the nucleus.

### Brefeldin A (BFA) treatment

Bombarded onion epidermal cells were incubated in 50 μg/ml Brefeldin A (BFA; Sigma) for 30 min at room temperature before observation under a confocal microscope [71].

### Protein assay

Protein concentrations were determined as described elsewhere [72] and using bovine serum albumin as standard.

### Reductase activity assay

The ability of the soluble NaTrxh mutants to reduce insulin disulphide bonds was evaluated as previously described [7] and compared with the reductase activity of the wild-type recombinant NaTrxh [22]. In these assays, 2.5 μg of purified Trx protein were used.

### Protein gel blot analysis and immunostaining

Proteins were fractionated by 12.5 % SDS-PAGE, blotted onto nitrocellulose, and then immunostained with polyclonal anti-GST (1:10,000 dilution), anti-NaTrxh (1:1,000) [22], or anti *S*_105_-RNase (1:10,000 dilution) [29].

### Affi-gel affinity columns and pull-down assays

Purified recombinant GST fusion proteins (30 mg) were immobilized on Affi-gel-10 (Bio-Rad), following the manufacturer’s instructions. The NaTrxh_rec_-Affi-gel affinity column used was the same as previously reported [22].

Protein crude extracts (1 mg) from *N. alata S*_105_*S*_105_ were obtained in a binding buffer (BB: 50 mM Tris–HCl, pH 7.9) and passed over each GST-NaTrxhAffi-gel column. After recovering the unbound fraction, ten bed volume washes were done with the BB. The column was sequentially washed as follows: (a) BB plus 1 % Tween-20, (b) BB plus 0.1 M NaCl, and (c) BB plus 0.2 M NaCl where indicated. Tightly bound proteins were eluted with a 50 mM glycine plus 50 mM NaCl, pH 2.6 buffer. The samples were neutralized by the immediate addition of 1 M Tris. Fractions were concentrated by cold acetone precipitation and analyzed by SDS-PAGE and western blot, as described above.

### Multiple alignment analysis

Amino acid sequences of plant Trxs were aligned by ClustalW [73]. The GenBank accession numbers used are as follows: from Trx h subgroup 1, *A. thaliana* (S58118 and S58119), *Brassica napus* (Q39362), *B. oleracea* (P68176), *N. tabacum* (Q07090 and P29449), and *Oryza sativa* (D26547); and from Trx h subgroup 2, *A. thaliana* (AAD39316, AAG52561 and S58123), *Ipomoea batatas* (AY344228), and *N. alata* (DQ021448).

### Modeling of the *N. alata* h2 thioredoxin

BLAST sequence analysis revealed h2 thioredoxin from barley (*H. vulgare*; PDB id 2IWT) as the closest homologue to the *N. alata* thioredoxin NaTrxh. With Modeller 9v4 [74,75], several possible alignments were then used to produce up to 100 initial homology models of NaTrxh. After evaluation using ANOLEA, the best models were recombined in Modeller, and the selection repeated. The best model was then used for a Molecular Dynamics (MD) simulation run at 35 ns in an octahedral water box (1.2 nm distance from the walls to the protein) with 0.15 M NaCl, at 303 K (Berendsen thermostat, with velocity rescaling) and standard pressure (Berendsenbarostat), using GROMACS v4.5 [33] and the GROMOS G53a6 force field [76]. After a 30 ns simulation, the radius of gyration and root mean squared deviation (rmsd) changes of the protein reached a plateau, and only the first 30 and the last 20 amino acids appeared partially unfolded. The most representative conformer was recovered with clustering from 30 to 35 ns, minimized, and the MD simulation repeated. Again, after clustering from 30 to 35 ns and minimization, a final model was produced. The last 5 ns of MD simulation were used to estimate the local pre-residue fluctuations (rmsf) as a tool to identify poorly structured regions in the protein. For this particular case, the repeated MD simulations converged better to a stable model than the classic simulated annealing MD. The reason behind this was not pursued further.

The final model was minimized again using the ROSETTA relax-fast protocol [77] and its quality evaluated. The ANOLEA total energy [37] is −1184.276 E/κT units, with positive energies (sum 41.691 E/κT units) in 12 of 52 residues (Figure 7). The ProQ scores [38,78,79] are LG 2.910 (very good) and MaxSub 0.301 (fairly good). MaxSub has been compared to other scores and was found to be a reliable quality score for homology-based models [79]. The biological appropriateness of the final model was rated using the Rd.HMM protocol [39]. The search of amino acid sequences compatible with the final model on the NCBI sequence databases retrieved 62 sequences. The top score corresponded to the NaTrxh amino acid sequence (GenBank accession AAY42864.1, HMM score 124, E-value 6.6 × 10^−31^). The original X-ray template is at position 29 with an HMM score of 20. In the remaining sequences, 45 were Trxh sequences, 15 lacked functional annotation, and none were annotated with an alternative function. The ratio of the HMM score to the NaTrxh sequence length is 0.84, which is indicative of a very good model [39]. The HMM alignment is consistent with an optimal threading of the amino acid sequence to the NaTrxh 3D model. From these data, the 3D coordinates of the NaTrxh predicted model could be rated as highly appropriate to host the NaTrxh amino acid sequence. In contrast to other quality assessment methods for protein structural models, the Rd.HMM protocol does not give false positives; therefore, the model may be regarded as a highly reliable prediction.

### Disordered regions of NaTrxh

We used DisEMBL [44,80] to predict, *in silico*, intrinsically disordered regions (IDRs) in the NaTrxh amino acid sequence. DisEMBL reports three different index values for assignment of IDRs: loops/coils, hot loops, and remark 465. Loops/coils indicate residues found within loops that are not necessarily disordered; hot loops indicate highly dynamic loop regions that should be regarded as disordered; and remark465 labels the Protein Data Base (PDB) records with missing coordinates in the X-ray structure, accordingly. The DisEMBL index was trained with PDB data to predict highly mobile regions likely to produce a poorly defined electron density in the would-be X-ray data [80].

### Immuno-gold and transmission electron microscopy

*N. alata* flowers were emasculated 48 h before anthesis and then collected as they reached maturity. Tissue was cut 2 mm below the stigma and fixed in 3% paraformaldehyde and 0.5% glutaraldehyde in 0.1 M PBS for a minimum of 4 h at 4°C. Tissue was rinsed in 0.1 M PBS three times for 10 min. The fixed tissue was dehydrated in a series of ethanol concentrations (30%, 40%, 50%, 60%, 70%, 80%, 90%, 96%, 100%) and infiltrated with LR-white resin by dipping it into solutions of increasing concentration (25%, 50%, 75%, 100%). The selected regions were then cut into ultra thin sections and placed in nickel grids. For immunolocalization, sections were blocked with TBST buffer (20 mM Tris pH 7.6, 150 mM NaCl, 20 mM sodium azide, 1% Tween 20, 5% BSA) for 1 h at room temperature and then incubated with a rabbit anti-NaTrxh antibody (1:10) at 4°C overnight. The sections were washed three times with TBST for 5 min and then incubated with 25 nm gold-labeled anti-rabbit IgG (1:10) for 2 h at room temperature. Grids were washed three times in TBST and then two times in deionized water. The sections were stained with uranyl acetate followed by lead citrate. Grids were observed and photographed with a JEOL 1200EXII electron microscope.

## Abbreviations

Trx: Thiorredoxin; Trx h: Thioredoxin type *h*; ER: Endoplasmic reticulum; ECM: Extracellular matrix; CW: Cell wall; BFA: Brefeldin A; DTT: Dithiothreitol; MD: Molecular dynamic; PDB: Protein data bank; IDRs: Intrinsically disordered regions; IDPs: Intrinsically disordered proteins; SI: Self-incompatibility/self-incompatible; GFP: Green fluorescent protein; SRK: S-locus receptor kinase; ORFs: Open reading frames; rmsd: Root mean squared deviation; SRP: Signal receptor particle; VMD: Visual molecular dynamics.

## Competing interests

The authors declare that they have no competing interests.

## Authors’ contributions

AAC and JAJD generated the molecular constructs and performed the molecular and transient expression assays. RRS, AZC and LPMC performed the structural *in silico* analysis. CEBA worked on onion epidermal cells transfection. CPIS performed the electron microscopy immune-localization experiments. YCZ generated and provided Nap11 constructs. JMG supervised the experiments. FCG supervised the research, designed the experiments and was involved in data analysis. FCG and JAJD wrote the manuscript. All authors read and approved the final manuscript.

## Supplementary Material

Additional file 1: Figure S1 The N- and C-terminal extensions in NaTrxh. Protein alignment of various plant Trxs h. NaTrxh N- and C- terminal extensions are bolded. The N-terminal extension was split into two subregions based on the Hidden-Markov-predicted cleavage site: Nα covers from the Met-1 to the Ala-16 residues; the Nβexpands from Ala-17 to Pro-27. Click here for file

Additional file 2: Figure S2 Hydrophobicity profiles of the N-termini of Nap11 and NaTrxh proteins. Dotted line: Nap11; solid line: NaTrxh. Click here for file

Additional file 3: Figure S3 A similar Nβ motif from *N. alata* (Nala [DQ021448]) is found in *Glycine max* Trxh1 and Trxh2 (Gmax [TRX1] and Gmax [TRX2], respectively), which are associated with the plasma membrane. The Nβ motif (underlined), essential to lead NaTrxh secretion, is conserved in Trxh1 and Trxh2 (both associated to the plasma membrane) from soybean [60]. Click here for file
