# Supplementary figures and images for: A novel motif in the NaTrxh N-terminus promotes its secretion, whereas the C-terminus participates in its interaction with S-RNase in vitro
Source: BMC Plant Biol. 2014 May 28;14:147. doi: 10.1186/1471-2229-14-147 (PMC4065587; doi:10.1186/1471-2229-14-147)

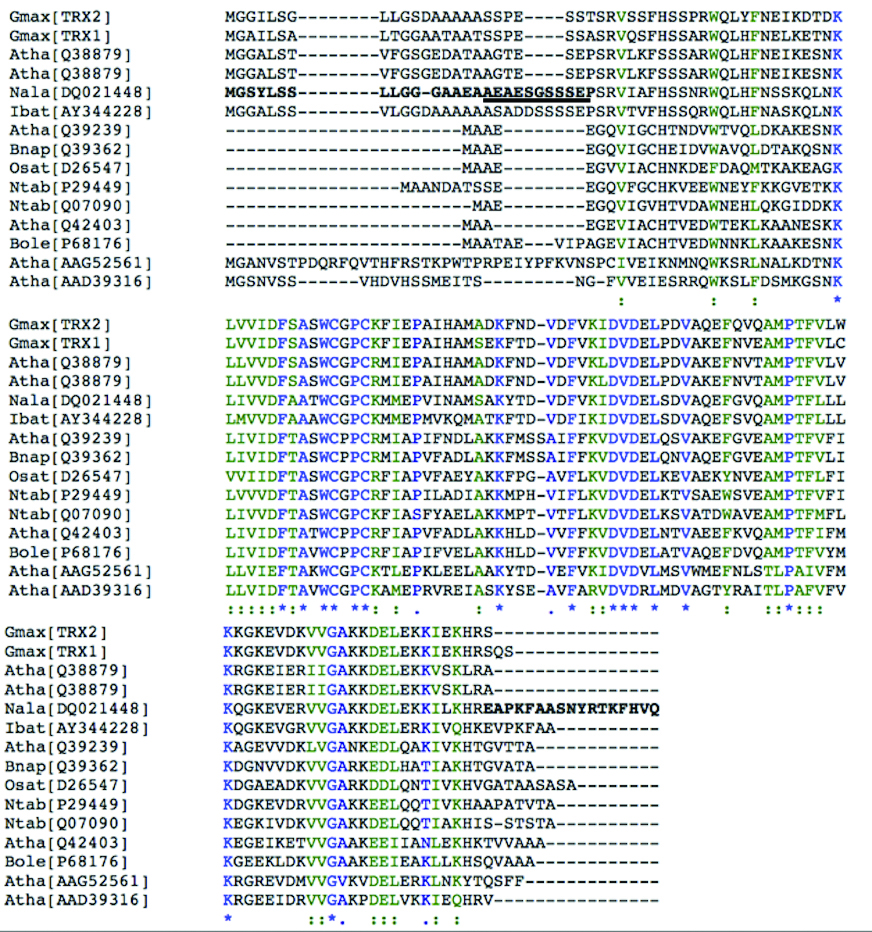

Supplement: Additional file 1: Figure S1 — The N- and C-terminal extensions in NaTrxh. Protein alignment of various plant Trxs h. NaTrxh N- and C- terminal extensions are bolded. The N-terminal extension was split into two subregions based on the Hidden-Markov-predicted cleavage site: Nα covers from the Met-1 to the Ala-16 residues; the Nβexpands from Ala-17 to Pro-27. [file 1471-2229-14-147-S1.jpeg]

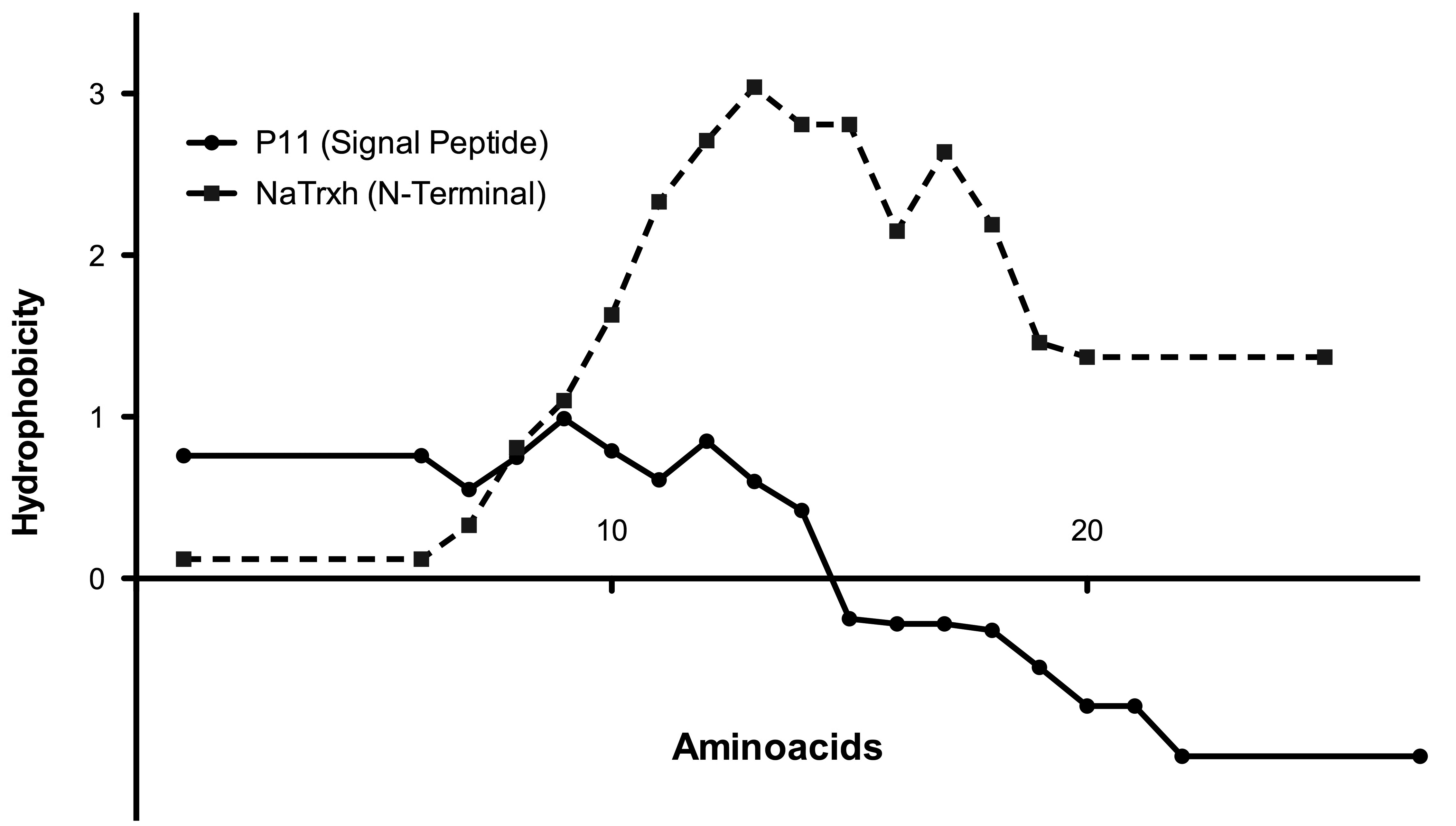

Supplement: Additional file 2: Figure S2 — Hydrophobicity profiles of the N-termini of Nap11 and NaTrxh proteins. Dotted line: Nap11; solid line: NaTrxh. [file 1471-2229-14-147-S2.jpeg]

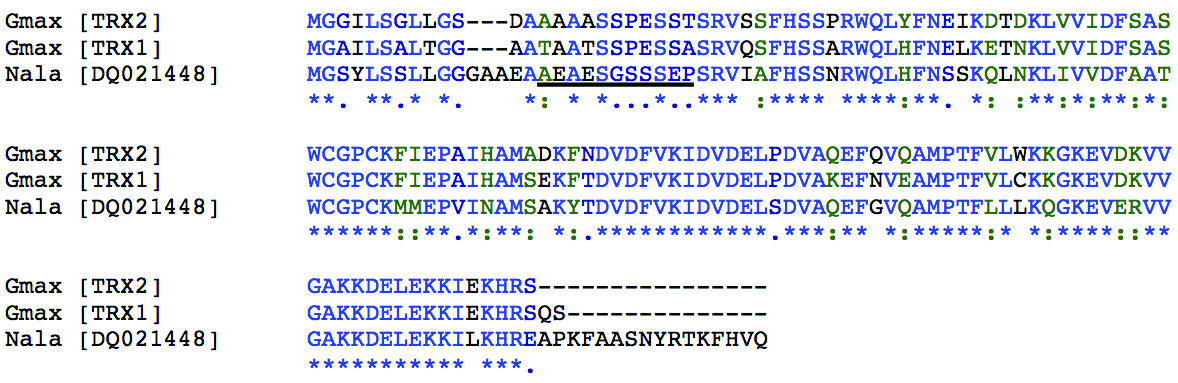

Supplement: Additional file 3: Figure S3 — A similar Nβ motif from N. alata (Nala [DQ021448]) is found in Glycine max Trxh1 and Trxh2 (Gmax [TRX1] and Gmax [TRX2], respectively), which are associated with the plasma membrane. The Nβ motif (underlined), essential to lead NaTrxh secretion, is conserved in Trxh1 and Trxh2 (both associated to the plasma membrane) from soybean [60]. [file 1471-2229-14-147-S3.jpeg]
